# Pulmonary Metastasectomy for Colorectal Cancer: Recent Reports Prompt a Review of the Available Evidence

**DOI:** 10.1007/s11888-014-0234-5

**Published:** 2014-08-27

**Authors:** Tom Treasure

**Affiliations:** Clinical Operational Research Unit, University College London, London, WC1H 0BT UK

**Keywords:** Colorectal cancer, Pulmonary metastasectomy, Carcinoembryonic antigen, Randomised controlled trials

## Abstract

Pulmonary metastasectomy for colorectal cancer is commonplace surgery, but the practice has grown on the basis of follow-up studies. These studies base their conclusion on the effectiveness of metastasectomy on the survival rates at 5 years of very highly selected patients. Three publications in the last year, a registry study, a meta-analysis and a randomised controlled trial of monitoring and early detection of cancer recurrence, prompted a review of the evidence. A critical examination of the evidence suggests that much of the apparent benefit may be due to selection of patients most likely to survive on the basis of well-known prognostic features, explicitly stated in the clinical record. Clinicians also assess their patients over time and do not offer surgery to those with faster progression. Such clinical judgements are of their nature often subtle and undocumented and thus cannot be retrieved from the clinical record. Although some patients may have long survival following pulmonary metastasectomy, and indeed their survival might be believed to be due to resection of pulmonary metastases, how many patients must be operated on to find these survivors? What is the number ‘needed to treat’? It may be that of the patients having metastasectomy, for the greater proportion it does not materially alter their survival. A randomised controlled trial to resolve this uncertainty is in progress. The Pulmonary Metastasectomy in Colorectal Cancer (PulMiCC) trial is recruiting in Britain and Europe.

## Introduction

After potentially curative surgery for colorectal cancer, a policy of monitoring patients for asymptomatic recurrence is the standard of care [1, 2]. Detection of recurrence is accomplished by measurement of carcinoembryonic antigen (CEA) and imaging with whole-body CT. This permits early detection of liver and or lung metastasis so that they can be evaluated for resection with intention to cure.

In 2007, the present author, who was receiving an increasing number of referrals to perform surgery for which there was no good evidence, challenged the effectiveness of pulmonary metastasectomy [3]. At about the same time, the European Society of Thoracic Surgeons (ESTS) opened its Lung Metastasectomy Project. When the Working Group reported in 2010, its leaders concluded ‘the level of evidence to support current practice is too low to set firm recommendations to the members of ESTS. In the absence of a randomized controlled trial looking at the effectiveness of pulmonary metastasectomy on survival and quality of life, it is unlikely that the current practice will ever be influenced.’

This led to the Pulmonary Metastasectomy in Colorectal Cancer (PulMiCC) randomised controlled trial (RCT), now recruiting in Britain and Europe [4].

Three articles published within the last year underline in different ways the continuing and now pressing need for better evidence.

1. A systematic review of 25 studies published from 2000 to 2011 provides a meta-analysis of 2,925 patients who had pulmonary metastasectomy. The three well-established prognostic factors (more than one metastasis; an interval since primary resection of under 2–3 years; elevated CEA level) each approximately double the likelihood of early progression of disseminated disease after pulmonary metastasectomy [5••].
2. A population-based study of 543 patients, estimated to include about 60% of patients having pulmonary metastasectomy in Spain in a 2-year period from 2008 to 2010, provides the first prospective study of practice [6••]. Of these patients, 45% had multiple metastases, half had intervals of under 28 months and CEA level was elevated in 46%. Many of these patients are therefore in the categories where the disease will progress irrespective of the metastasectomy.
3. An RCT involving 1,202 patients, the Follow-up After Colorectal Surgery (FACS) trial, found that intensive CEA and/or CT monitoring does detect metastatic cancer earlier than minimum follow-up, but the ensuing surgery did not result in any increased survival compared with that of patients in whom the metastatic disease remained undiscovered [7••].

Wide acceptance that metastasectomy is effective in improving survival is based on numerous follow-up studies of highly selected patients. Randomised trials, control data and intention-to-treat analysis are lacking. An analysis of the how oncologists and surgeons arrived at their belief in the effectiveness of metastasectomy is the subject of this review.

## The Evidence for Pulmonary Metastasectomy in Colorectal Cancer

There must now be over 100 follow-up studies, and they are still being submitted for publication. Amongst them there have been numerous multivariate analyses, including the landmark report of the International Registry of Lung Metastases [8]. There were three systematic reviews of pulmonary metastasectomy in colorectal cancer between 2007 and 2010 [9–11]. They are mutually consistent in their findings and are consistent with the most recent systematic review and meta-analysis reported in 2013.

In the meta-analysis, four factors were associated with shorter survival time after lung metastasectomy:

1. For patients with multiple lung metastases compared with a solitary metastasis, there was a hazard ratio (HR) of 2.04 [95 % confidence interval (CI) 1.72–2.41]. Having more than a solitary metastasis doubles the likelihood of dying within 5 years.
2. Elevated prethoracotomy CEA level nearly doubles the likelihood of dying within 5 years (HR 1.91; 95 % CI 1.57–2.32).
3. A ‘disease-free’ interval shorter than 2–3 years between the primary operation and lung metastasectomy increases by about 60% the likelihood of dying within 5 years. (HR 1.59, 95 % CI 1.27–1.98).
4. Positive hilar and/or mediastinal lymph nodes also increase the likelihood of dying within 5 years by about 60% (HR 1.65, 95 % CI 1.35–2.02).

No doubt aware of the potential for confusion, the *Journal of Thoracic Oncology* published a biostatistics primer for ‘what clinicians ought to know’ setting out the difference between prognostic and predictive factors: ‘A prognostic factor is a variable that is assessed before starting any treatment; based on the value of this factor, the clinician can expect that a patient may have a better or worse clinical outcome (such as survival or response), regardless of what treatment the patient receives’ [12].

It is important to remember that the first three factors are general prognostic factors, as is the third if the information is available before the metastasectomy [12].

It is impressive that these factors remain so powerful in the analysis of Gonzalez et al. [6••] because these have been well known for many years and operated-on patients were highly selected in the full knowledge of these factors. For them to emerge from an analysis with these HRs suggests that they are commonly overridden, as they were in the Spanish study [6••]. The authors of the meta-analysis, in their discussion, take the view that ‘as long as a R0 resection is feasible, it seems currently unfair to deny surgery for those patients with two to four lesions’, which reflects current beliefs in practice rather than being a conclusion that can be derived from the data [5••].

The fourth factor, the evidence of further dissemination from the lung metastasis to hilar and mediastinal nodes, is in these series usually a pathology finding because it would be unlikely and indeed irresponsible to knowingly operate on such patients. Metastasectomy is likely to be ineffective if patients have already further dissemination via the pulmonary lymphatics. The operation is doomed to fail in its objective to achieve R0 resection. Although it has been proposed that mediastinoscopy should precede pulmonary metastasectomy [13], we cannot know how many patients have mediastinal staging investigations because reports are always of patients who have had metastasectomy, and they do not tell us anything of the selection process.

Many studies go as far as to exclude patients who did not have histological confirmation of R0 resection [9, 10], thus further defeating any attempt to understand the outcomes on the basis of ‘intention to treat’, which is the information that should be put to patients when metastasectomy is being discussed with them. This illustrates one of the many pitfalls in the custom and practice of surgical follow-up studies as a means of reporting results of surgery and the impossibility of drawing reliable inferences from them [14].

Amongst the systematic reviews was a quantitative synthesis of 3,504 patients in 51 studies reported from 1971 to 2007 [11]. This revealed that that although the practice had grown, the case mix had been remarkably consistent over about four decades. In round figures, 60% of metastasectomy operations were for a solitary metastases, the interval averaged 36 months and 60% of patients had died within 5 years.

## Pulmonary Metastasectomy as Palliation

There are few data on symptoms in any of these follow-up studies. [11]. Most patients having pulmonary metastasectomy are asymptomatic with respect to colorectal cancer. Occasionally, a pulmonary metastasis may cause symptoms (cough, haemoptysis, pain, etc.), and then surgery is assessed on an individualised basis.

At the terminal phase of the disease, there is not usually a pulmonary component to the symptoms or mode of death which might have been prevented by surgery. The policy of seeking out and resecting metastases is therefore not palliative in any sense.

## Selection or Surgery as the Determinant of Survival

Reporting a small comparative study of pulmonary metastasectomy in 1980, Åberg et al. [15] wrote: ‘It has been assumed, implied, or claimed that the 5-year survival without operation is nil. Control material is, however, lacking.’

Both sentences remain true 34 years later: it is widely believed that any 5-year survivors after pulmonary metastasectomy can thank the operation for their survival, but there are no control data. The best estimates of the degree of selection in the Spanish study [6••] is that these are fewer than 5% of metastatic colorectal cancer patients, and estimates from Italian and Japanese studies indicate that the rates may be around 2–3%, but the appropriate denominator is hard determine.

The assumption that survival at 5 years, were it not for lung metastasectomy, approaches zero is false. Data from the Thames Cancer Registry show that for patients with metastases *at presentation* (stage 4 in its terminology), the 5 and 10-year survival rates were 10% and 5%, respectively. We can consider the implications of these data in selection. We have known for years the characteristic of patients who have longer survival after metastasectomy. Clinicians caring for these patients have had, in addition, ample opportunity to review imaging to look at the number of metastases and their appearance and growth over time. So patients in follow-up studies were not only selected on the basis of the explicit criteria retrievable from the records but were further selected in full knowledge of their progression over time. This is probably the most powerful determinant of whether they will be alive some years later, and it might well be that the selected minority of patients will contain the natural survivors. A selection of 100 of 1,000 patients might include the 50 destined to live longer, and the resulting 50% survival is thus easily explained. We cannot capture this process from follow-up studies, and we must surely realise that the gap between survival without and with lung metastasectomy is not the difference between 0 and 50%.

It should also be remembered that the process of selection has as its precondition that the patient must have survived to the time point of being considered for metastasectomy and therefore includes immortal time bias [16–18]. This is particularly true if, for example, responsiveness to chemotherapy is included in selection, and is particularly illustrated by apparently favourable results of second and third metastasectomy. There is a large conditional component of having already survived to the time point for a patient to be included in the cohorts having repeated metastasectomy.

The Thames Cancer Registry data were used to model what might have been the 5-year survival rates for patients in two large follow-up studies [19, 20]. Survival curves were constructed for registry patients with a similar mix of cancer stages at registration and who remained alive for a period similar to the ‘disease-free interval’ in the published follow-up studies. The two 5-year survival rates were similar [21]. For more readily accessible accounts of this study, see the subsequent articles in which it is used [22, 23] (Table 1).

**Table 1:** Reported and modelled 2-year survival

Authors	Date	Patients	Reported 5-year survival rate (%)^a^	Modelled 5-year survival rate (%)
McCormack et al. [19]	1992	144	40 (32-48)	55
Okumura et al.[20]	1996	159	41 (33-48)	50

A mathematical modelling study [21] in which Thames Cancer Registry survival data were used to construct survival analysis for groups of patients with a Dukes stage similar to that of patients in the surgical follow-up studies and still alive at 36 months, which was the average interval from primary to metastasectomy operation to exclude patients who had not survived long enough to have become candidates for pulmonary metastasectomy

^a^The 95 % confidence interval is given in *parentheses*

How can this be? From their recollections, clinicians defend their practice in the belief that formerly no such patients survived beyond 1 year or so and that now they see long-term survivors. It would not be the first time that the clinical impressions of committed and dedicated clinicians are not supported by evidence when they are put to a fair test [24]. Patients are selected for surgery on the basis of many factors, including the trajectory of their clinical state over a period, a feature that is not captured in surgical follow-up studies. The authors rely on the limited information recorded at a point in time when the decision was taken to perform metastasectomy. Back at the time of selection, the rate of progression is evident to clinicians, and those patients progressing are unlikely to appear in the operating theatre.

## How the Literature and the Belief in It Grew

In the course of our studies of pulmonary metastasectomy, we became aware of the fascinating method of citation network analysis [25]. Greenberg [25] proposed that ‘citation can be used to generate information cascades resulting in unfounded authority of claims’.

The citation lists of the 51 follow-up studies used in the quantitative synthesis [11] were used to analyse the nature of the evidence on which those authors themselves had relied in writing up their follow-up studies [26].

**Fig. 1:**
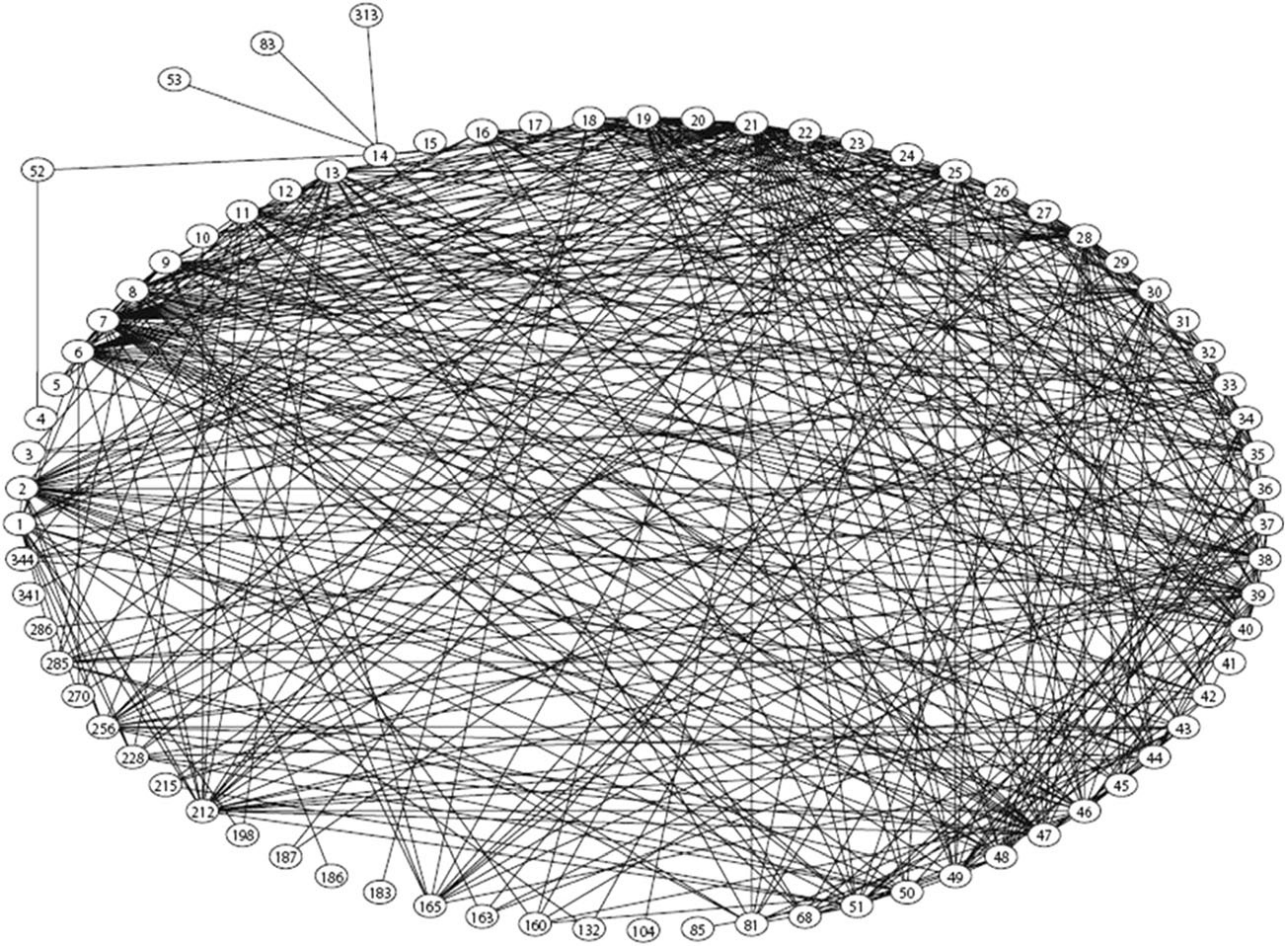
This graphic is from a network analysis in which nodes *1*–*51* are the follow-up studies of lung metastasectomy for colorectal cancer in the quantitative synthesis, and the remaining nodes, to a total of 72, are colorectal cancer studies which are cited. The article by Åberg et al. [15] questioning the effectiveness of lung metastasectomy is cited only twice, whereas there appears to be a feeding frenzy of mutual citation amongst the believers. (From Fiorentino et al. [26], with permission from Nature Publishing Group)

What we discovered was frenzy of mutual citation amongst authors with shared beliefs. (Fig. 1). We confirmed Greenberg’s statement that ‘unfounded authority was established by citation bias against papers that refuted or weakened the belief; amplification, the marked expansion of the belief system by papers presenting no data addressing it; and forms of invention such as the conversion of hypothesis into fact through citation alone.’

The article by Åberg et al. [15] formally challenging the belief in metastasectomy reporting the only, albeit small, comparative study was cited twice. A case report buried within a 1944 ‘state of the art’ lecture to the Boston Medical Society concerning progress in thoracic surgery [27] was cited by 14 of 51 of the index papers, and is the thirteenth most frequent of 334 cited papers. The article by Åberg et al. is directly relevant, and would show up in any search including the word ‘metastasectomy’. The article by Blalock [27] has no relevance to the practice and can only have been passed on from author to author; it would not have been found in a literature search. There is no clue in the title, and the tenuous connection with practice would take some perseverance to discover if indeed the article had been read at all. The cascade effect is ‘like rolling a snowball: it gets bigger and bigger – but it is just more snow’ [26].

The practice of monitoring patients by means of their CEA level grew in the 1970s and 1980s, and was the basis of an RCT to determine if the earlier detection that it allowed, before the presence of symptoms, would result in better survival. The study recruited patients from 1982 to 1993. There was no benefit, but instead a small *excess* of deaths in those randomised to have CEA monitoring prompted second-look surgery (91 of 108 versus 88 of 108; difference 2.8%) [28]. The reporting of the results of the trial lapsed in 1994, perhaps perceived to have been overtaken by events. As part of the initiative to restore invisible and abandoned trials [29, 30], the full results have been published [31]. The FACS trial asked the same question and returned the same answer in 2014. For 901 patients randomised to have CEA monitoring, CT or both, the mortality was 18.2%, whereas for those having symptom-prompted investigations and clinical follow-up, the mortality was 15.9%, a difference of 2.3%, similar to that found in 1994 favouring patients spared unavailing second-look surgery (Table 2).

**Table 2:** How the evidence for metastasectomy for colorectal cancer was overtaken by events: the history of adoption of liver resection for colorectal metastasectomy is documented by Grunhagen et al. [37]

Date	Publication
1954	Wangensteen et al. [38] advocated second-look surgery in asymptomatic patients following colorectal cancer
1971–1978	Resection of recurrent cancer after potentially curative resection of colorectal cancer was believed sometimes to lead to ‘cure’ [39–41]
1974–1980	CEA monitoring was shown to detect asymptomatic recurrence of colorectal cancer following surgery, with the possibility of better results for second-look surgery [42–46]
1981	NIH consensus call for a trial of CEA monitoring [47]
1982	The CEA Second-Look trial started recruiting [31]
1982–1989	Hughes et al. [48–51] published registry results of liver resection with increasing numbers
1990–1991	Scheele et al. [52–54] published a growing institutional cohort of liver resections
1992	McCormack et al. [19] published 10-year results of pulmonary metastasectomy in colorectal cancer
1992	Rosen et al. [55] published the power calculation for a randomised trial of liver resection. Claims for benefit versus natural history were so far apart that, if correct, 36 patients would have been sufficient to prove it
1994	CEA Second-Look Trial results available [28, 56]
1994	Stangl et al. [57] wrote ‘benefit … has been clearly demonstrated’
1994	Scheele et al. [58] wrote ‘trials on … effectiveness of hepatic resection for metastatic colorectal cancer [would be] not only obsolete but unethical’
1997	The International Registry of Lung Metastases reported its analysis of prognostic factors for lung metastasectomy [8]

*CEA* carcinoembryonic antigen, *NIH* National Institutes of Health

## Oligometastasis and Ablative Therapies

Minimally invasive therapies such as radiofrequency ablation (RFA) and stereotactic radiotherapy can destroy tissues accurately and reliably without the need for open surgery. They have a very valuable role in treating symptomatic lesions where the evidence of effectiveness is basically the N of one trial. In comparison with surgery, they are proposed as an alternative to metastasectomy, being safer and less invasive. However, the arguments rest on a prior belief that surveillance and eradication of asymptomatic metastases improves survival, which has been shown not to be the case [7••]. If we are unsure of the effectiveness of surgery in this regard, effectiveness of less invasive ablation must be tested in its own right. This has been done for colorectal liver metastases. There was no difference in survival between those patients who had and those patients who did not have RFA in this RCT [32•]. On the basis of the only RCT evidence available, RFA was thus ineffective in improving survival.

The oligometastatic state, a term coined by radiation therapists [33] and now in the argot of cancer teams and tumour boards, has shaky evidence. It is a concept without empirical evidence. The ‘fewness’ of metastases is no doubt a reflection of the biological nature of that individual’s cancer: the more metastases, the more aggressive the cancer, and the converse is a necessary corollary. There is no suggestion that the distribution of the number of metastases is bimodal, which would be expected if the oligometastatic state were a pathological entity. It is no more than an arbitrary convention to give some identity to what is no more than a therapeutic opportunity [34]. To define a disease by the therapy available and that would be needed to treat it is perfectly reasonable and is standard, for example, in end stage renal disease [35, 36], but the treatment should still be proven to be effective.

## Conclusions

We know that of all patients having pulmonary metastasectomy, most go on to die of their colorectal cancer. Of all patients with pulmonary metastases, only 2-3% have pulmonary metastasectomy, and of this highly select group, authors further select subsets in whom there were high 5-year survival rates. But how many are ‘cured’? Or is it that this process of selection on selection simply identifies the minority with slowly progressing disease?

It is the view of the author that pulmonary metastasectomy is offered to patients without adequate evidence. There are many historical precedents for reversals in practice and belief. The PulMiCC RCT is in progress and will provide the first controlled data to guide future practice.

The PulMiCC trial design is based on the premise that of patients with lung-only or liver and lung metastases many will be not be offered metastasectomy. At the other end of the spectrum of adverse to favourable features are the patients that most teams will consider for metastasectomy. It follows that if there is a ‘no’ for some and ‘yes’ for others, there must be a transitional zone of uncertainty. It is only in that zone, and it varies between the more conservative and the more aggressive team, that randomisation is invited in the second stage of the recruitment process. However, all patients can be recruited into the first stage.
